# Meta-analysis of the clinical and laboratory parameters of SFTS patients in China

**DOI:** 10.1186/s12985-016-0661-9

**Published:** 2016-11-29

**Authors:** Miao-miao Liu, Xiao-Ying Lei, Xue-jie Yu

**Affiliations:** 1 School of Public Health, Shandong University, 250012 Jinan, China; 2 Department of Pathology, University of Texas Medical Branch at Galveston, Galveston, TX 77555-0609 USA

**Keywords:** Severe fever with thrombocytopenia syndrome (SFTS), Case fatality rate, Clinical manifestation, Risk factor

## Abstract

**Background:**

Severe fever with thrombocytopenia syndrome (SFTS) is an emerging hemorrhagic fever in East Asia, which is caused by a novel bunyavirus-SFTSV. Many studies have reported the clinical characters of SFTS patients, but the reports were not consistent and a systematic summary of clinical manifestations and laboratory parameters are not available.

**Method:**

A comprehensive literature research of Web of Science, PubMed, Wan Fang Data, and Chinese National Knowledge Infrastructure databases was conducted on articles which have described the clinical characters of SFTS patients. Data from selected studies were pooled by using STATA VERSION 12.0 software.

**Result:**

Nine articles comprising 844 laboratory-confirmed SFTSV cases were included in this meta-analysis. The pooled case fatality rate was 16% (95% CI: 0.13–0.19). The major clinical characters of patients with SFTSV infection were fever, thrombocytopenia, leucopenia, gastrointestinal symptoms, and central nervous system manifestations. The risk factors for severe disease included bleeding tendency, central nervous system manifestations, elevated serum enzymes, and high viral load. Although there is no specific antiviral therapy for SFTSV infection, symptomatic treatment and supportive therapy including intensive monitoring is the most essential part of case management.

**Conclusion:**

The major clinical characters of patients with SFTSV infection were fever, thrombocytopenia, leucopenia and gastrointestinal symptoms, and central nervous system manifestations. The risk factors for severity and fatality among SFTS patients included: old age, CNS manifestations, bleeding tendency, elevated serum enzymes, and high vial load.

## Key points

Severe fever with thrombocytopenia syndrome is a severe hemorrhagic fever without effective treatment. Patients from endemic areas with fever, thrombocytopenia, leucopenia, gastrointestinal symptoms, and central nervous system manifestations should be considered as SFTS.

## Background

Severe fever with thrombocytopenia syndrome (SFTS) is an emerging hemorrhagic fever, which was first discovered in rural areas of eastern and central China in 2009 [1] and more recently in South Korea and Japan [2, 3]. SFTS is caused by a novel bunyavirus-SFTS virus that has been detected from ticks and ticks are thought to be the vector of SFTSV [1, 4].

The major clinical symptoms of SFTS patients included acute fever (temperature of 38 °C or more), thrombocytopenia, leucopenia, gastrointestinal symptoms, and central nervous system (CNS) manifestations, followed with multiple organ dysfunctions [5–8]. Some cases in critical condition had the following manifestations: disturbance of consciousness, gastrointestinal bleeding, pulmonary hemorrhage, and skin bruising. The cases died due to diffuse intravascular coagulation (DIC), multiple organ failure, and shock [7–9].

Previous studies have confirmed that the outcome of the SFTS patients has been associated with the levels of their clinical characters and the biochemical markers at the early stage [5–8, 10]. As the fatality rate of this disease was surprisingly high and the main risk factor for this phenomenon was not clear, this study was designed to analyze the relationship between various factors and the outcome of the SFTS patients through meta-analysis and to predict the severity of the disease.

## Methods

### Search strategy

We searched Web of Science, PubMed, Wan Fang Data, and Chinese National Knowledge Infrastructure databases from 2009 to 2016 using the following terms: (“SFTS” OR “SFTSV”) and “patient”. Moreover, we not only identified articles through database retrieval, but also by reviewing the reference list of retrieved articles to search for further relevant documents.

### Inclusion and exclusion criteria

Articles included in this meta-analysis had to meet the following criteria: the first and foremost, the SFTS patient mentioned in the selected studies must be confirmed as meeting one or more of the following criteria: (1) isolated the virus from the patient’s samples, (2) SFTSV RNA was detected from the patient’s serum by a quantitative reverse-transcriptase polymerase chain reaction (qRT-PCR) and (3) a 4-fold or greater increase of antibody titers was detected between a paired serum samples of the patient collected from the acute and convalescent phases of infection; Secondly, the article contained the most recent or largest population was selected when the studies using the same or overlapping data by the same authors. Exclusion criteria included small scale studies with fewer than 15 patients, works designated as case reports, conference abstract, letters, editorials or reviews, and duplicated publications.

### Data extraction and quality assessment

Based on the aforementioned inclusion and exclusion criteria, the preliminary screening was made by reading the title and abstract of the literature. Then, after reading the full text, the documents were eventually confirmed. All selected articles were independently screened and evaluated by two reviewers. After cross-checking, any disagreement was resolved by a third evaluator. The following information was extracted from every eligible article: the first author, year of publication, country, number of patients, number of fatalities, and the positive number of each clinical character.

### Statistical analysis

I-squared was chosen to reflect the heterogeneity among studies. Values of *p* < 0.05 and I-squared >50% were considered to be statistically significant. According to the result of heterogeneity, the proper model was adopted to merge the clinical parameters of the patients: When the data were homogeneous, the fixed effect mode was used and for heterogeneous data, the random effect model was employed. Publication bias was assessed by using Egger’s test: *p* < 0.05 was considered to be statistically significant. For each selected publication, the pooled fatality rate, pooled positive rate of each clinical character, and their 95% confidence intervals (CI) were calculated. We also calculated simple pooled rates along with a naive weighted average method [11]. All analyses were performed with STATA 12.0 (Stata Corp LP, College Station, Texas, United States).

## Results

### Literature search

A total of 241 relevant articles were retrieved after the preliminary screening from the electronic databases and other sources. Two hundred one of these literatures were excluded after review of the title and abstract for irrelevant topics, and an additional six documents were removed for duplication of the data. After reading the full text of the remaining 34 articles, 25 articles were excluded due to lack of some indicators. Nine studies were included for further meta-analysis. The data from these studies included in the meta-analysis was shown in Table 1.

**Table 1:** Summary of the studies included in the meta-analysis

Reference number	Author	Publication year	Country	Samples
[1]	Yu et al.	2011	China	154
[5]	Deng et al.	2013	China	115
[6]	Gai et al.	2012	China	59
[7]	Sun et al.	2012	China	59
[8]	Ding et al.	2014	China	59
[9]	Liu et al.	2013	China	311
[10]	Zhang et al.	2012	China	49
[32]	Cui et al.	2013	China	16
[33]	Wen et al.	2014	China	22

### Basic information description

A total of nine articles were included in this study. From the nine eligible studies, a total of 844 patients were included in this meta-analysis, which were published from 2011 to 2015. All the individuals were laboratory-confirmed as SFTS patients. In all SFTS patients, the proportion of female was 48.1%. The patients ranged from 7 to 89 years of age and most of them (80%) were over 50 years old. Ninety-two percent of the patients were farmers living in wooded and hilly areas and working in the fields before onset of the disease. Most confirmed SFTS patients (96%) involved in this analysis were admitted to the hospital from May to October.

### The case fatality rate and clinical character of SFTS patients

**Fig. 1:**
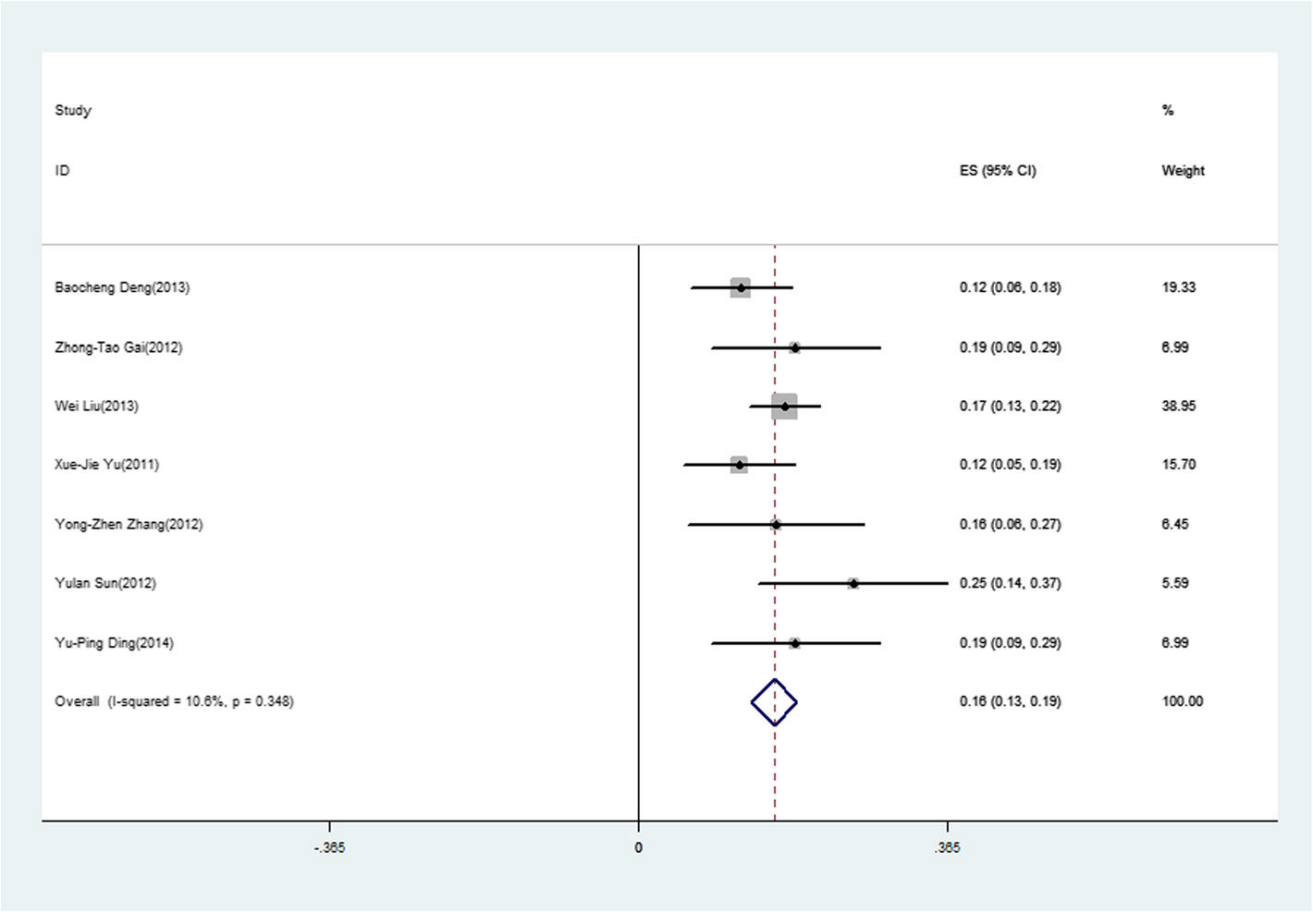
Forest plot of case fatality rate for SFTS patients. The point estimates from each study were shown as *solid squares*. The pooled estimates were shown as an empty diamond. Error bars represented 95% CIs. (Reference No. [32] and No. [33] were excluded because they did not mention the fatality rate)

The forest plot of case fatality rate of SFTS patients was shown in Fig. 1. The pooled case fatality rate was 16% (95% CI: 0.13–0.19). By heterogeneity analysis, I-squared of case fatality rate was 10.6% (*p* = 0.348), implying that there was no significant heterogeneity among the samples.

The pooled positive rates of clinical characters are shown in Table 2. As the definition of ‘fever’ was different in various studies, the heterogeneity of pooled positive rate of fever was 85.2% (*p* < 0.05), it was recommended to choose the simple pooled rate to describe the situation of fever in SFTS patients. The simple pooled positive rate of fever was 94%. As the I-squared of pooled rate of thrombocytopenia was 71% (*p* = 0.03), we chose the simple pooled rate to reflect the combined effects. Symptomatic patients comprised 65% of the group. All the studies have defined ‘leucopenia’ as leukocyte count <4.0 × 10^9^/L, and 88% of patients presented with this laboratory finding. Patients with SFTSV infection usually have gastrointestinal symptoms, including anorexia, nausea, vomiting, diarrhea or abdominal pain. The polled positive rate of those symptoms were 83%, 54%, 39%, 34%, 22%, respectively. In addition, patients with SFTSV infection also had hemorrhagic manifestations and central nervous system manifestations (Table 2).

**Table 2:** The pooled positive rates of clinical characters^a^

			Pooled	95% CI		Simple pooled
	I^2^ (%)	*p*	Rate	Low	Up	Rate
Fever^b^	85.20	<0.05	0.99	0.97	1.00	0.94
Leucopenia^c^	0.00	0.70	0.88	0.82	0.94	0.73
Thrombocytopenia^d^	71.00	0.03	0.83	0.51	1.16	0.65
Anorexia	58.70	0.09	0.83	0.76	0.91	0.85
Weakness	46.70	0.09	0.74	0.54	0.94	0.83
Nausea	34.60	0.21	0.54	0.49	0.60	0.55
Myalgias	49.60	0.09	0.51	0.42	0.60	0.62
Proteinuria	62.30	0.10	0.50	0.39	0.61	0.51
Lymphadenopathy	61.80	0.05	0.39	0.29	0.49	0.35
Vomiting	55.50	0.05	0.39	0.33	0.46	0.38
Joint pain	0.00	0.33	0.35	0.27	0.43	0.28
Chills	30.10	0.24	0.32	0.22	0.43	0.27
Diarrhea	66.60	0.02	0.31	0.23	0.39	0.34
Hematuria	25.10	0.25	0.29	0.22	0.36	0.29
Cough	67.60	0.08	0.28	0.20	0.36	0.26
Abdominal pain	26.80	0.25	0.22	0.15	0.29	0.29
Dizziness	0.00	0.53	0.20	0.16	0.24	0.25
Headache	19.80	0.29	0.17	0.13	0.21	0.22
Case fatality rate	10.60	0.35	0.16	0.13	0.19	0.16
Petechiae	16.70	0.31	0.08	0.06	0.11	0.11

^a^The data would be excluded if it did not mention the corresponding clinical character

^b^Fever was defined as body temperature ≥38 °C in references [1, 5, 32, 33]; ≥37.5 °C in references [8, 9]; ≥39 °C in reference [6]

^c^Leucopenia was defined as leukocyte count <4.0 × 10^9^/L

^d^Thrombocytopenia was defined as platelet count <150 × 10^9^/L in references [5, 32, 33]; <100 × 10^9^/L in references [1, 8–10]

### Laboratory parameters of SFTS patients

During the early stage of SFTSV infection, the serum viral load was high both in non-fatal and fatal patients. Nevertheless, along with the development of the disease, the serum viral load decreased in non-fatal patients, but still remained at a high level in fatal cases. All the selected literatures showed that SFTSV infection was associated with liver and kidney function impairment. Laboratory examination found an increase of serum enzymes, such as aspartate aminotransferase (AST), alanine aminotransferase (ALT), lactate dehydrogenase (LDH), creatine kinase (CK), as well as creatine kinase MB fraction (CK-MB), which indicated the liver dysfunction. The level of serum enzymes increased to a peak about 10 days after onset in non-fatal SFTS cases, and then gradually declined to normal level, however, it progressively increased in fatal cases. Similar to the above findings, blood urea nitrogen (BUN) also increased in SFTS patients, which is a parameter that can indicate impairment of renal function. Furthermore, in all detected clinical laboratory parameters, blood coagulation times (activated partial thromboplastin time, APTT and thrombin time, TT) were longer among SFTS patients than the healthy individuals. In addition, cytokine levels, such as interleukin-6 (IL–6), IL-8, IL-10, granulocyte colony-stimulating factor (G-CSF), and interferon-γ (IFN-γ) were enhanced in SFTS patients and were significantly higher in fatal cases than the non-fatal individuals.

## Discussion

Most cases are farmers (92%), who were living or working in wooded and hill areas, where ticks were commonly found. This phenomenon indicated that ticks are most likely to be the vector of SFTSV [1, 4, 12]. Most patients were admitted to hospital from May to October. During this period, farmers were engaged in the agricultural activities, and were easily exposed to ticks. This interpretation indirectly confirmed that ticks are the vector of SFTSV. In addition, it has been confirmed that SFTSV can be transmitted to humans by contact with blood or body fluid from SFTS patients [13, 14]. Therefore, individuals should not only pay attention to reduce exposure to ticks in daily life, but also pay attention to personal protection when they take care of SFTS patients.

The disease mainly occurs in the elderly population [15, 16]. Consistent with the aforementioned results, in this study, the vast majority of the patients are over 50 years old. Studies revealed that older age was a significant risk factor for fatal outcome of SFTS patients [8, 9, 17, 18]. The cause of this phenomenon-older patients tended to have more severe symptoms, is likely to be that the older people have lower immunological capacity to limit the replication of SFTSV. The high incidence of SFTS in old people could be caused by a population skew toward to old people in countryside because many young people left rural area to work in city. However, a previous study indicated the SFTSV antibody positive rate was not significantly different among people at different age groups in rural area, suggesting that age is the critical risk factor to determine for SFTS morbidity and mortality [19].

The typical features of SFTS are acute fever and thrombocytopenia. About 94% of patients had acute fever at the early stage and most of them returned to normal in 10 days. At the early stage of infection, as exogenous stimuli, the virus activated the body to release endogenous pyrogens. Endogenous pyrogens, in turn, circulate to the thermoregulatory center of the brain where it cause an elevation of the body temperature [20]. Over 65% of patients had a lower platelet count (platelet count <150 × 10^9^/L). Both in vitro and in vivo assays confirmed that the virus adhered to platelets and facilitated the phagocytosis of platelets by macrophages [8, 21]. Those patients with thrombocytopenia often accompanied with hemorrhagic tendency, such as petechiae and microscopic hematuria. In addition, severe hemorrhagic symptoms, such as gastrointestinal bleeding, macroscopic hematuria and hematoma, are commonly observed in sever patients with disseminated intravascular coagulation [3]. In comparison with SFTS patients who survived, the deceased patients had a more serious hemorrhagic tendency. It suggests that bleeding tendency was associated with the death of SFTS patients.

The central nervous system symptoms commonly occurred approximately 5 days after the onset of illness, and persisted for 1–2 weeks [6]. The CNS manifestations of SFTS patients included dizziness, headache, apathy and coma before death. The CNS manifestations of deceased patients were more serious than survivors indicated that CNS manifestations are the risk factors for the death of SFTS patients.

In SFTS patients’ serum, the levels of liver, kidney, and cardiac enzymes are commonly dramatic elevated, indicating the impairments of those organs. In survivors, the serum enzymes reached to the maximum levels in about 10 days after infection, and then gradually declined to the normal levels. However, in deceased patients, the levels of serum enzymes appeared to progressively rise and reached to the maximal values before death. The risk analysis revealed that elevated AST, LDH, CK and CK-MB were risk factors associated with severity among SFTS patients and fatality among severe SFTS patients [5, 6].

Levels of serum cytokines such as IL-6, IL-10, G-CSF, and IFN-γ were increased in SFTSV-infected patients. Clinical studies have indicated that cytokine storm, characterized by overproduction of certain cytokines, is commonly associated with the severity of the disease [5, 7]. Consistent with the findings aforementioned, the virus infection including hemorrhagic fever virus, such as, Crimean Congo Hemorrhagic Fever (CCHF), Ebola, and Rift Valley fever virus can lead to cytokine storm which is associated with the progression of the disease [22–24]. Cytokine storm occurring in the acute phase of the disease has been widely hypothesized to be the main cause of morbidity and mortality for SFTSV infection [25]. Under normal circumstances, inflammatory cytokine and chemokine could promote activation and chemoattraction of lymphocytes and exert antiviral effects. However, recent findings indicated that cytokine storm, characterized by an overwhelming and imbalanced profile of cytokines, could become excessive and harmful [26].

Serum viral load was considered to be the major laboratory marker for clinical diagnosis. After the onset of the illness, the initial serum viral load was high in all patients. Then the virus was gradually cleared in survivors at the recovery phase, but still remained high in the fatal patients. There was a difference between the serum viral load of the surviving and deceased patients [8, 27–29]. High serum viral load was considered to be the high risk factor that resulted in the death of SFTS patients [10]. Therefore, it was identified as an important parameter to predict the outcome of the SFTS patients. Meanwhile, studies founded that there was a positive correlation between the serum viral load and the levels of various cytokines. In other words, during the acute phase of SFTSV infection, the higher viral load, the higher levels of cytokines (except for RANTES and PDGF-BB) [7, 28]. These findings suggested that the high viral load may lead to excessive secretion of cytokines, which could further aggravate the progression of the disease.

Ribavirin is reported to be effective for treating CCHF infections and hemorrhagic fever with renal syndrome [30, 31]. However, studies have found that intravenous ribavirin had no effect on reducing the serum viral load in SFTS patients [5, 9]. Meanwhile, the use of tetracycline for the treatment in SFTS patients is not justified unless co-infection can be confirmed [32]. In addition, treatment of SFTS patients with corticosteroids for acute respiratory distress syndrome is controversial. This is because though corticosteroids can suppress the cytokine storm, it also can increase the risk of developing critical disease from viral infection [5]. Hence, supportive therapy is the most essential and effective part of case management [33].

Our meta-analysis has some limitations: the primary limitation of our meta-analysis was that there are subtle differences in the definition of the clinical characters in different studies. Since in different articles, the forms of the laboratory parameters were not uniform, we could not carry out a systematic analysis for these indicators. Also, like most other meta-analysis, the overall findings from the meta-analysis were limited by the quality of the primary studies.

## Conclusion

In conclusion, the major clinical characters of patients with SFTSV infection were fever, thrombocytopenia, leucopenia and gastrointestinal symptoms, and central nervous system manifestations. The risk factors for severity and fatality among SFTS patients included: old age, CNS manifestations, bleeding tendency, elevated serum enzymes, and high vial load.
